# Children’s Online Collaborative Storytelling during 2020 COVID-19 Home Confinement

**DOI:** 10.3390/ejihpe11040115

**Published:** 2021-12-10

**Authors:** Cristina Alonso-Campuzano, Giuseppe Iandolo, María Concetta Mazzeo, Noelia Sosa González, Michelle Jin Yee Neoh, Alessandro Carollo, Giulio Gabrieli, Gianluca Esposito

**Affiliations:** 1 Department of Psychology, School of Biomedical Sciences, European University of Madrid, Calle Tajo S/N. (Urb. El Bosque), Villaviciosa de Odón (Madrid), 28670 Madrid, Spain; cristina.alonso@psisemadrid.es; 2 Observation and Functional Diagnosis Division, PSISE Clinical and Developmental Psychological Service, Calle Albendiego 7, 28029 Madrid, Spain; concetta.mazzeo@psisemadrid.com (M.C.M.); noelia.sosa@psisemadrid.com (N.S.G.); 3 Psychology Program, Nanyang Technological University, Singapore 639818, Singapore; michelle008@e.ntu.edu.sg (M.J.Y.N.); giulio001@e.ntu.edu.sg (G.G.); 4 Department of Psychology and Cognitive Science, University of Trento, 38068 Trento, Italy; alessandro.carollo@studenti.unitn.it (A.C.); gianluca.esposito@ntu.edu.sg (G.E.)

**Keywords:** storytelling, collaborative learning, social distancing, COVID-19, cognitive development

## Abstract

Digital collaborative storytelling can be supported by an online learning-management system like Moodle, encouraging prosocial behaviors and shared representations. This study investigated children’s storytelling and collaborative behaviors during an online storytelling activity throughout the 2020 SARS-CoV-2 home confinement in Spain. From 1st to 5th grade of primary school, one-hundred-sixteen students conducted weekly activities of online storytelling as an extracurricular project of a school in Madrid. Facilitators registered participants’ platform use and collaboration. Stories were audio-recorded, transcribed verbatim, and analyzed using the Bears Family Story Analysis System. Three categories related to the SARS-CoV-2 pandemic were added to the story content analysis. The results indicate that primary students worked collaboratively in an online environment, with some methodology adaptations to 1st and 2nd grade. Story lengths tended to be reduced with age, while cohesion and story structure showed stable values in all grades. All stories were balanced in positive and negative contents, especially in characters’ behavior and relationships, while story problems remained at positive solution levels. In addition, the pandemic theme emerged directly or indirectly in only 15% of the stories. The findings indicate the potential of the online collaborative storytelling activities as a distance-education tool in promoting collaboration and social interactions.

## 1. Introduction

Storytelling is a relevant educational tool to develop competencies to attribute meaning, to communicate, and to share personal experiences [1,2,3,4,5,6]. It is a socio-cognitive ability that allows individuals to express their inner world and share representations, memories, and experiences with a conversational interlocutor [5,7,8,9]. Storytelling involves defining individual identity and attributing meanings to experiences [5,10,11], which starts to develop in early childhood from symbolic play [12], language [13], and interpersonal relationships [14].

From the preschool stage, typically, developing children build their storytelling skills in interactions with peers and with the help of adults who encourage the production of coherent, understandable, and relevant stories to be heard [15,16]. Children under three years old tell primitive stories with word concatenations without continuous action sequences or causal connections between events [17,18]. From the age of three, oral narrative language begins to develop [19]. Between three and six to seven years, there is the first stage of development of narrative cohesion, which transitions from a description of actions individually to a story that includes thematic sequences and reactions to events [2,20]. From the age of seven/eight, there is a second evolutionary leap in the ability to create a story that includes problematic elements and solutions, mediated by events that facilitate or complicate the story’s progression [6,7]. From the age of twelve, throughout adolescence and early adulthood, a linguistic-narrative and social development is achieved. In this period, it is necessary to define personal identity, through relationships with equals, with parents, and in complex social realities such as higher education and work [21,22,23].

Regarding the evolution of storytelling content, there is a typical tendency, especially after six to seven years, in children without emotional and behavioral difficulties, to balance negative and maladaptive contents with positive and adaptive ones [1,2]. As in the decision-making process, the general plans necessary to create a story are used to control the declared information, allowing the construction of new global meanings and progressively closing the elements opened throughout the story [24,25,26].

In educational contexts, storytelling encourages narrative thinking, creativity, problem-solving abilities, expressive-linguistic skills, socialization, collaboration, and inclusion of students with special-educational needs and behavioral difficulties [27,28,29,30,31]. In teaching environments, it is becoming increasingly common for stories to be used to work on emotional and psychological contents, presenting characters who experience situations like those of the audience [32] to promote social cognition, empathy [4,33,34,35,36], and coping skills [32]. Collaborative storytelling activities add interpersonal aspects to the experience and elaboration of content since there are multiple opportunities for personal expression and social interaction [37,38] while working on story creation and development. Moreover, peer collaboration increases motivation, self-expression, the questioning of ideas, reflection, and elaboration of content through a common language [39,40,41,42].

Digital storytelling is a powerful educational tool that enhances the possibilities in creating shared stories and their subsequent transmission [28,30,43,44]. It results from the combination of collaborative storytelling and multimedia technology [45,46,47] Online learning-management systems such as Moodle (https://moodle.org (accessed on 20 November 2021)) can support digital storytelling through the creation of creative and collaborative communities where students are the protagonists and where there is sharing of knowledge and narratives through collaboration [48,49,50,51]. Barriers to online learning are perceived to decrease as age and experience increase [52,53]. In online cooperative digital storytelling, the class can be involved in real-time interactions, showing horizontal and networking characteristics, where students are also responsible for their peers’ creative collaboration skills [48,54,55]. Online creative collaboration involves socio-cognitive skills such as communication, interaction, reflection, exploration of possible options, reliability, and mutual co-operation. In addition, real-time interaction is one of the advantages of an online-learning system like Moodle that can be modularized with different digital extensions for free discussion and meetings, collecting opinions, sharing, and writing documents, ideas, and evaluations.

In March 2020, the World Health Organization declared COVID-19, a disease caused by SARS-CoV-2, a pandemic; therefore, governments imposed restrictions to control the epidemiological situation. During the first wave of the pandemic, Spain was one of the most-affected countries, with home confinement from March to June. Starting from 13 March 2020, the government prohibited leaving home and ordered the closure of all non-essential services, manufacturing activities, shops, sports, artistic events, and schools. This situation had negative consequences for mental health in the general population [56,57,58,59] and for families, students [60,61,62,63,64], and teachers [65]. In particular, the lockdown as a result of the 2020 SARS-COV2 pandemic forced changes and adaptations in routines, teaching, and work methodology, with many students still unfamiliar and unaccustomed to online interactions, especially in primary school [66]. Social interactions are relevant in learning, especially in kindergarten and primary school [67,68,69,70,71]. Therefore, the effects of the 2020 school closings had to be addressed with educational practices aimed to support social contact and the performance of shared activities, as is usually done in schools, while also considering inclusion of students with special-educational needs within the group.

The activity proposed in this article is an online collaborative storytelling project that combines aspects of storytelling, collaborative learning, and technology to carry out a fun activity with peers during the pandemic home confinement.

This study aimed to analyze the effect of the activity on students’ collaborative storytelling competencies, collaboration during the task, and the degree to which the COVID-19 theme appeared as a spontaneous theme in groups’ stories, as a sign of concern about the pandemic and the lockdown situation.

The research design is cross-sectional and explored the group narratives of Spanish children aged between 6 and 11 years old, participating in an online storytelling activity during SARS-CoV-2 home confinement from April to June 2020. The research objectives were to describe and analyze participants’ behaviors during the online collaborative activity, group-story form and content, and references to the COVID-19 pandemic. Four hypotheses were explored:

**Hypothesis** **1** (**H1**). *Primary school students can work collaboratively within an online space for group-storytelling activities.*

**Hypothesis** **2** (**H2**). *The formal complexity of collaborative stories evolves with students’ age according to the school grade.*

**Hypothesis** **3** (**H3**). *There is a balance between the positive and negative contents of the group stories.*

**Hypothesis** **4** (**H4**). *The COVID-19 theme appears in at least 50% of the stories, introduced spontaneously by the students.*

## 2. Materials and Methods

### 2.1. Procedure

The online collaborative storytelling activity was developed as an extracurricular project, between April and June 2020, during the lockdown and schools’ closure due to the SARS-CoV-2 pandemic health emergency in Spain. In collaboration with the teaching team of a school in Madrid, we offered all students from 1st to 5th grade the opportunity to participate in the activity, twice a week, in one-hour sessions. Volunteer child psychologists from a Madrid health center prepared and coordinated the different sessions.

The project was presented to the school’s families and teachers through a webinar meeting, providing a session schedule per grade and documentation for voluntary registration. The activity was free, and the data for registration were collected guaranteeing the participants’ anonymity according to European (EU GDPR 2016/67) and Spanish (Law 3/2018) privacy standards.

**Figure 1:**
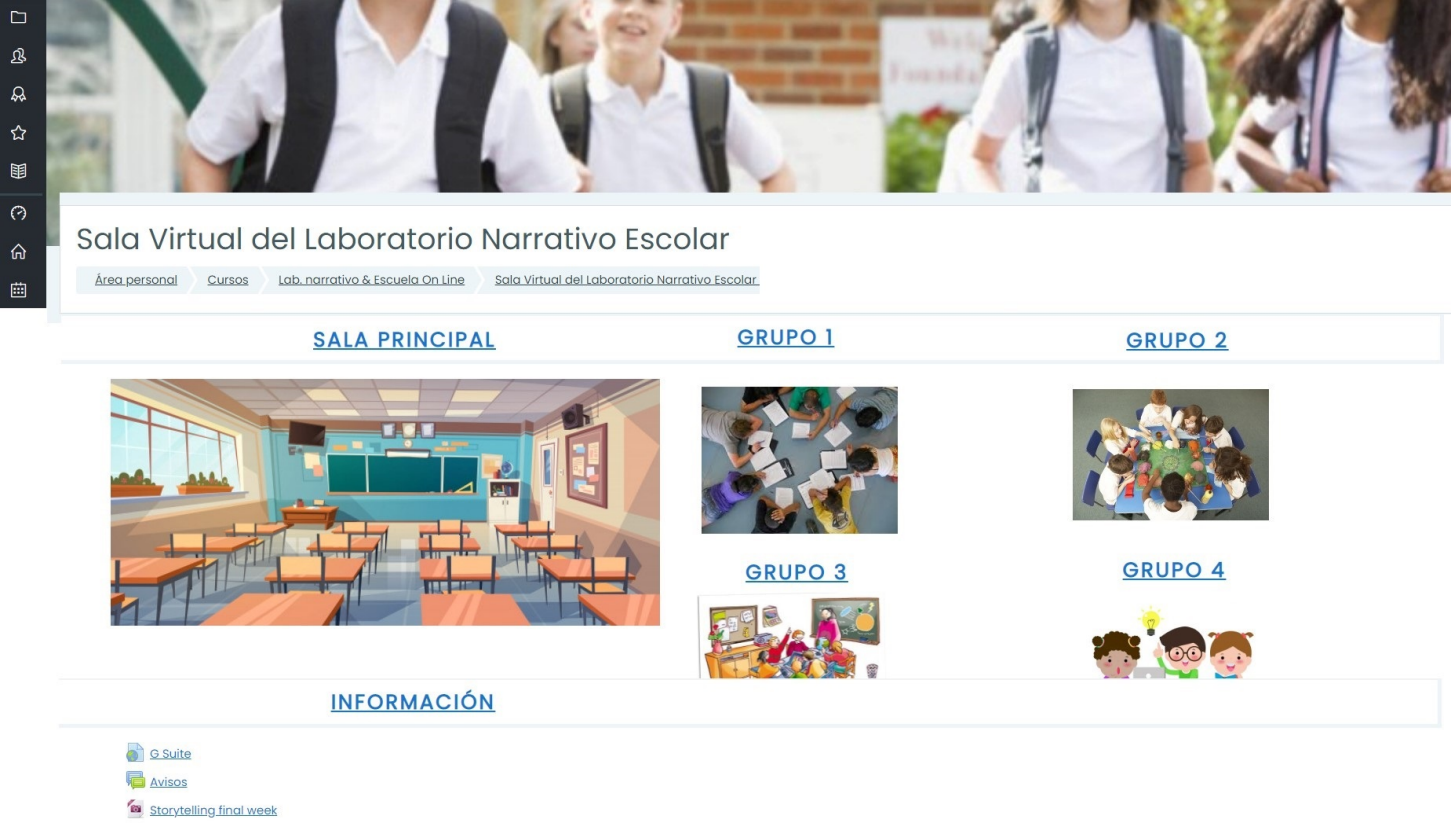
Home page of the online platform for the collaborative storytelling activity.

The online platform was developed in “Moodle 3.8+”, through which registered students could access the activity through a personalized user account. The platform had links to five Google Meet video-conference rooms, in which students and facilitators carried out the collaborative storytelling activity following a schedule organized by school grades (Figure 1). The platform also had a forum for families and students, where facilitators published summaries of each session, and it was open to comments and questions.

Parental informed consent was obtained from all subjects involved in the study before gaining access to the online-platform activities. Participants could not access the activity without accepting the privacy policy and providing the parental-informed research consent.

Each session followed the same protocol with six phases: presentation, explanation of the session’s topic, group formation, group discussion, storytelling to the class, and scoring of prosocial behaviors. The phases are as follows:

- Presentation: the facilitator introduces himself/herself to the class in the main room of the platform and invites each participant to greet each other.
- Explanation of the session’s topic: the facilitator explains the activity and presents the session topic through a situational image on the topic using the screen-sharing function, saying: “It is time to tell a story. Think about how you can create a story about the shared image on the screen. In this image, we can see *…*” (the facilitator describes the image in detail). There is a different topic for each session. The facilitator also reminds participants of the essential parts of a story (beginning, middle, and end).
- Group formation: the facilitator forms small groups randomly and refers the students to the different video-conference rooms marked with a number on the platform (Figure 1). Sharing their screen, the facilitator explains where they should go and that, once they finish their story, they should return to the main room. In each video-conference room, groups met with a facilitator who supports them during the task and who begins the activity with the following words: “Now you can talk with your group-mates and think about how you could tell a story. In the end, I will also ask you to choose a representative of the group to tell the story to the other groups.”
- Group discussion: each group has 15 min to discuss the topic and create the story; when they have agreed, they should return to the main room, where they meet with the rest of the groups.
- Storytelling for the class: after each group returns to the main video-conference room, the facilitator that awaits them says, “Well, it is time to tell your stories. Has each group chosen its representative? If you have not done so, do it now to hear each group’s story.” All group representatives explain the story to the class in a maximum time of 5 min.
- Designation of prosocial behavior-related scores: the last 10 min of the session are dedicated to a debriefing phase and to reward students through a digital-token economy system (https://classdojo.com (accessed on 20 November 2021)). We considered only positive social behaviors.

The themes were chosen from previous sessions of collaborative face-to-face narrative laboratories conducted in the primary school on emotional, relational, and inclusion themes. The facilitator introduced to participants a stimulus image that represented the situation, followed by a title. During the presentation phase, the facilitator described the picture and the title of the session.

There were thirteen session themes proposed by the facilitators: 1. “Family,” 2. “Happiness,” 3. “Anger,” 4. “Sadness,” 5. “Fear,” 6. “Surprise,” 7. “Disgust,” 8. “Inclusion,” 9. “Lies and truths,” 10. “Friendship,” 11. “Love,” 12. “Empathy,” and 13. “Conflict.”

The themes were chosen from previous experiences of collaborative face-to-face narrative laboratories in the primary school on emotional, relational, and inclusion themes. The facilitator introduced to participants from a stimulus image that represented the situation, followed by a title. During the presentation phase, the facilitator described the picture and the title of the session.

After the first week of activity, we revised the 1st- and 2nd-grade sessions’ protocol since most students did not have the essential digital autonomy to perform the initially proposed changes between video-conference rooms. For this reason, the protocol was adapted with the younger participants, creating working groups that had to develop a “chained story.” In this methodology, facilitators asked participants to create a collective story, where each participant introduced a phrase or episode sequentially, following what was said by the previous classmate.

The facilitators audio-recorded and transcribed verbatim all the group stories for subsequent analysis through the Integrated Analysis System of the Bear Family Projective Test [72], with specific content variables related to COVID-19 added ad-hoc for this study’s purposes. Two judges, blind to the study’s hypotheses, coded the stories independently. The reliability was evaluated in 45% of the stories analyzed using Cohen’s kappa index, which was statistically acceptable (Kappa = 0.93). The facilitators also made observations for each session through an observation sheet in which they had to rate groups’ behaviors and collaborative work. An independent observer carried out a second assessment of the group’s behavior and collaborative work in this case. The inter-observer agreement analysis was evaluated in 22% of the sessions, obtaining a statistically acceptable Cohen’s kappa index (Cohen’s kappa 0.89).

Each participant received a final virtual diploma and a compilation of each course’s stories in the last activity session. Finally, some stories were presented in a closing webinar meeting with the families.

### 2.2. Instruments

The stories developed by the participants were analyzed according to the Integrated Analysis System of the Bear Family Projective Test [72], taking into consideration the stories’ form (number of words, propositions and episodes, structure, and narrative cohesion) and content (problems, relationships, and character’s behaviors). The Bears Family Integrated System is the story coding system of The Bears Family Projective [72] developed to capture the essential characteristics derived from language, words, episodes, behavior, and character relationships in children’s stories between three and eleven through a standard evaluation protocol. Variables for the formal factor of the story and the content factor of the story are defined as:

Formal Factor of the Story

- Number of words: number of the story words.
- Propositions: number of basic units that declare a story’s action.
- Episodes: number of events made up of propositions connected through subordination or specification or divided by coordination.
- Narrative Structure: the narrative structure index refers to the typical elements that revolve around a story and shape it: 1. introductory phrase; 2. protagonist; 3. temporal setting; 4. environment setting; 5. conclusion; 6. long-term conclusion. In the integrated system, a point is assigned for each typical story element, allowing a variation of the index between zero and six.
- Narrative Cohesion: the story develops around a point of view, a theme, interconnected episodes, a plot, and one or more problematic events with a solution. The index can vary between zero and eleven.

Content Factor of the Story

- Problems with a solution: story episodes that contain a difficulty or danger for one or more characters, followed by a solution.
- Problems without a solution: story episodes that contain a difficulty or danger for one or more characters, without any solution.
- Positive relationships: story episodes with positive relationships between characters.
- Negative relationships: story episodes with negative relationships between characters.
- Characters’ behavior: story episodes with adaptive, aggressive, rule-rejecting, and guilt-ridden behaviors performed by the characters.
- Balance 1: number of positively vs negatively solved problems.
- Balance 2: number of solved vs not-solved problems.
- Balance 3: number of positive vs. negative relationships between characters.
- Balance 4: number of adaptive vs. non-adaptive behaviors of the characters (aggressive + rule-rejecting + guilt-ridden behaviors).

In addition, we added three content variables related to COVID-19 to the story analysis:

- Direct reference to COVID-19/pandemic: presence/absence of the words COVID-19, virus, or pandemic in the story.
- Indirect reference to COVID-19/pandemic (catastrophe): presence/absence of contents related to any kind of catastrophe in the story (e.g., the end of the world, a tsunami, a war, etc.).
- Indirect reference to COVID-19/pandemic (illness): presence/absence of contents related to any kind of illness in the story.

Facilitators observed the students’ platform use and collaboration during the task in each session through an observation sheet adjusted for the study, on a three-point Likert scale (1—no/never; 2—enough/sometimes; 3—a lot/always). The facilitators observed different items within two domains: (i) platform use and (ii) collaboration.

Platform Use

- Mute a partner: one or more participants turn off a partner’s microphone during the session.
- Interruptions: one or more participants do not respect conversation turn-taking and overlap in the conversation.
- Off-topic chat use: one or more participants use the chat on a different topic than the narrative activity.
- Group self-regulation: one or more members of the group call their classmates to order and concentrate on the task.
- Positive comments: one or more group members express positive comments about the contribution of partners.

Collaboration

- Focus on the task: the group works on the same assigned task. Both the topic(s) dealt with during the collaborative process and the form and content of the group’s result are taken into consideration.
- Social awareness: awareness of emotions and feelings of partners in the interpersonal relationship.
- Social cognition: ability to understand social cues and reciprocal relational behaviors (facial expressions, gestures, and actions).
- Social communication: ability to communicate ideas and feelings to others, following conversation’s rhythm and turn-taking.
- Social motivation: initiative and social exploration, start of social interactions, conversation, or reciprocal response-encouraging discourse.
- Space for everyone: the group makes space for all its members to expose their opinion, make contributions, ask questions, and comment on solutions.
- Inclusion: the group tries to adapt and commit to relational exchanges according to all its members’ needs.

In the Supplementary Materials, Tables S1 and S2 show two examples of the story analysis, collaboration, and platform-use results.

### 2.3. Participants

From first to fifth grade, one hundred sixteen students from a school in Madrid (Spain) participated in the online-storytelling activity. The sample consisted of 53 boys (45.7%) and 63 girls (54.3%), aged between 6.3 years and 11.2 years (mean 102.61 months; SD 19.06; Table 1). The largest number of participants belonged to the second grade (n = 39, 33.6%), followed by fifth (n = 31, 26.7%), first (n = 27, 23.3%), third (n = 12, 10.3%) and, finally, fourth grade (n = 7, 6%).

Of a total of 116 students, 8% (n = 10) manifested neurodevelopmental or functional difficulties, as indicated by families and teachers in the student’s registration sheet. There was at least one student with such difficulties per grade (Table 1).

### 2.4. Data Analysis Plan

First, the sample’s descriptive statistics, group composition, attendance, and study variables are presented, associated with the Shapiro–Wilks normality test for story analysis, platform use, and collaboration.

For Hypothesis 1, we presented the descriptive statistics based on the facilitators’ observation sheet about platform use and collaboration. Subsequently, Pearson’s r (for variables with normal distribution) and Spearman’s r (for variables with non-normal distribution were calculated, and Kruskal–Wallis tests were conducted to explore the possible effect of school grades on participants’ platform use and cooperation. Finally, Spearman’s r and Cronbach’s α coefficient were employed to explore the internal consistency of the collaborative categories.

For Hypothesis 2, regarding formal story complexity and school grade, Spearman’s r was calculated, and Mann–Whitney tests were conducted.

For the third hypothesis, descriptive statistics of the content balance indexes 1 and 2 (related to story problems), index 3 (character’s relationships), and index 4 (characters’ behaviors) were calculated. Subsequently, Mann–Whitney and Kruskal–Wallis tests were conducted to explore the differences in story balance indexes by school grade, the number of group members, the storytelling methodology (chained vs. small group), and the session topic. Lastly, we calculated the correlations between content-balance indexes and the formal aspects of the story using Spearman’s r.

For the last hypothesis, we calculated the frequencies of direct and indirect references to the COVID-19 pandemic in the stories related to the last hypothesis. Next, we explored school-grade differences, the number of group members, the storytelling methodology, and the session topic through Chi-squared and Kruskal–Wallis non-parametric statistics. Finally, we explored possible descriptive tendencies between the frequency of appearance of COVID-19-related topics during the three months of the online activity, from April to June 2020.

## 3. Results

A total of 71 sessions were held, with an average of 14 sessions per course (Mean = 14.2; SD = 2.39) between April and June 2020. Participants’ attendance ranged from a minimum of 28% of the sessions in the fifth graders up to 72% in second graders. Out of a total of 81 stories created by all courses, fifth graders created the most stories (29 stories; 35.8%), followed by second (20 stories; 24.7%), fourth (14 stories; 17.3%), first (12 stories; 14.8%) and, finally, third graders (6 stories; 7.4%). The working groups were mainly mixed in participants’ sex (77 stories, 95.1%), while there was a group of only girls (1.2%) in one session and groups of only boys (3.7%) in three sessions. Students with neurodevelopmental and functional difficulties created 15 stories (18.5%) together with their grade classmates without such difficulties. The activity was carried out satisfactorily in all the 71 sessions, producing 81 group stories, from a minimum of 2 to a maximum of 20 participants (mean = 6.43; SD = 2.63; Table 2). Differences in the number of participants in the groups were determined both by attendance and the story-creation methodology, where sessions with the chained-story methodology had larger groups of 1st- and 2nd-grade students.

Except for the number of words of the group story, the study variables showed a non-normal distribution (Table S3). For this reason, parametric statistics were used for the only variable with a normal distribution (number of words) and non-parametric statistics for the rest of the variables.

### 3.1. Hypothesis 1: Collaboration in Online Group Storytelling

**Figure 2:**
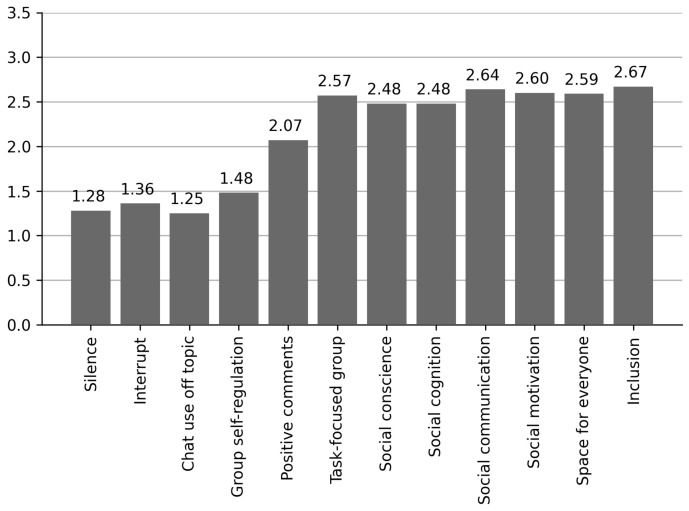
Platform use and collaboration mean scores (1. No/never; 2. enough/sometimes; 3. a lot/always; N = 81).

The results supported hypothesis 1 that primary school students would work collaboratively within an online space for group storytelling (Table 3, Figure 2). According to the facilitators’ observations, platform use and collaboration scores reached acceptable values in all sessions (Figure 2). Behaviors related to improper platform use (silence, interruption, off-topic chat use, and group self-regulation) were low. On the other hand, behaviors that indicate good platform use and collaboration reached medium-high values (positive comments, task-focused group, social awareness, social cognition, social communication, social motivation, space for everyone, and inclusion).

Despite finding differences between school grades (Table 4), the results showed no correlations between school grade, participant’s platform use, and collaboration (Table 3). On the other hand, the number of group members correlated negatively with the school grade (Spearman’s r = −2.6, *p* = 0.02).

Finally, all the collaboration variables positively correlated with each other (Table 4), reaching a high internal consistency (Cronbach’s α = 0.90).

### 3.2. Hypothesis 2: Formal Complexity of Stories across Grades

The results partially supported hypothesis two that formal complexity of collaborative stories evolve across school grades. The results indicate that participants in higher grades created shorter stories (words, Pearson’s r = −0.37, *p* < 0.01; propositions, Spearman’s r = −0.43, *p* < 0.01; episodes, Spearman’s r = −0.47, *p* < 0.01), while no significant correlations were observed between grade year, story cohesion (Spearman’s r = −0.40, *p* = 0.72), and story structure (Spearman’s r = 0.03, *p* = 0.80). It is related to the chained-stories methodology adopted for 1st and 2nd grade with a facilitator in each work group. The use of the chained-stories methodology is associated with an increased story length in terms of the number of words, propositions, and episodes.

### 3.3. Hypothesis 3: Balance of Positive and Negative Contents

The results supported hypothesis 3 that the stories will have a balance of positive and negative contents. All balance indexes showed variability (SD > mean), which was higher for indexes 3 and 4 (Table 5). Indexes 1 (Balance 1: positive vs negative problem solution) and 2 (Balance 2: solved vs unsolved problems), both related to story problems, showed a symmetrical and mesokurtic distribution (Balance 1: M = 1.32; SD = 1.90; Skewness = −0.32 (0.27); Kurtosis = −0.06 (0.53); Balance 2: M = 1.89; SD = 1.87; Skewness = −0.46 (0.27); Kurtosis = 1.82 (0.53)). Index 3 (positive vs negative character’s relationships) presents a symmetrical and leptokurtic distribution (Balance 3: M = 0.73; SD = 2.67; Skewness = −0.57 (0.27); Kurtosis = 4.06 (0.53), while index 4 (adaptive vs non-adaptive characters’ behaviors) shows an asymmetric negative leptokurtic distribution (Balance 4: M = 0.59; SD = 2.47; Skewness = −1.30 (0.27); Kurtosis = 3.07 (0.53). The results indicate a balanced proportion of solved problems, while characters’ relationships and behaviors present a greater variability between positive and negative contents, suggesting a good balance.

The results show no significant differences between school grade, the number of group members, or storytelling methodology (chained vs. small group) in any of the four content balance indexes of the story (Table 6).

**Figure 3:**
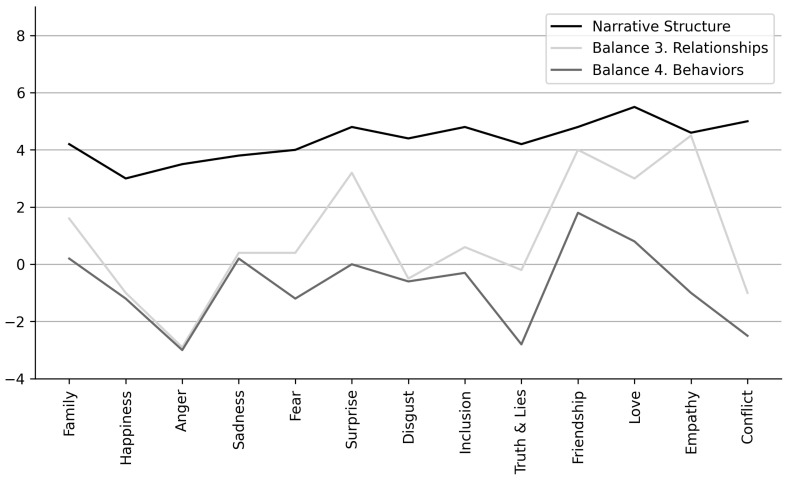
Mean scores in story variables with significant differences by sessions’ theme (narrative structure, Balance 3—positive/negative relationships, Balance 4—adaptive/non-adaptive behaviors)

Regarding the session topic, there were no significant differences in the two balance indices related to the problems in the story: (a) in index 1 related to the balance between story problems with positive and negative solutions (Balance 1; Kruskal–Wallis test, 16.99; df 12; *p* = 0.15); (b) in index 2 related to the balance of story problems, which were solved and unsolved (Balance 2; Kruskal–Wallis test, 17.00; df 12; *p* = 0.15). On the other hand, the results indicated significant differences (Figure 3): (a) in the balance index related to the positive and negative relationships between story characters (Balance 3; Kruskal–Wallis test, 39.28; df 12; *p* < 0.01); (b) between adaptive vs. non-adaptive characters’ behaviors (Balance 4; Kruskal–Wallis test, 22.24; df 12; *p* < 0.03); and (c) in story structure (Kruskal–Wallis test, 25.75; df 12; *p* = 0.01) but not in terms of narrative cohesion (Kruskal–Wallis test, 20.87; df 12; *p* = 0.52). Moreover, the results showed a correlation between the story’s narrative structure and the content balance index 3 (Spearman’s r = −0.32, *p* < 0.01) but not with the content balance index 4 (Spearman’s r = 0.12, *p* = 0.30).

### 3.4. Hypothesis 4: COVID-19-Related References in Stories

The results did not support hypothesis four that COVID-19-related references would be present in at least 50% of the stories. The results showed a considerably lower frequency of 14.81%. The groups introduced the COVID-19 topic only in 12 stories of the total of 81 prepared during the activity (Table 7), with five direct references to COVID-19 or the pandemic (6.17%), three indirect references in the form of catastrophe (3.70%), and four indirect references in the form of illness (4.93%).

There were non-significant differences in such COVID-19-related references between grades ($\chi^{2}$ (4, 81) = 4.91, *p* = 0.39; Kruskal–Wallis test H (4) = 4.85, *p* = 0.30), number of group members ($\chi^{2}$ (10, 81) = 14.21, *p* = 0.16; Kruskal–Wallis (10) = 14.04, *p* = 0.17), nor the methodology of elaboration of the stories (Chi squared $\chi^{2}$ (1, n81) = 0.50, *p* = 0.47; Mann–Whitney U = 747.00; *p* = 0.4). Although the relationship between the topic of the session and the number of references to the COVID-19 topic ($\chi^{2}$ (12, 81) = 14.46, *p* = 0.26; Kruskal–Wallis (12) = 14.67, *p* = 0.27) was not significant, there was a greater incidence of COVID-19 references in the sessions about fear (4 references in 10 sessions on this topic, 40%), followed by family (5 references in 14 sessions, 36%), lies and truths (1 reference in 3 sessions, 33%), disgust (1 reference in 8 sessions, 13%), and, finally, sadness (1 reference in 11 sessions, 9%). No direct or indirect references were observed in the stories created in the sessions with the other topics. Regarding the percentage of sessions with COVID-19-related references each month, we observed a decreasing trend between April and June 2020. In April, students made six references in a total of 27 sessions (22%); in May, we registered six references in forty-four sessions (13.63%), while in June, there were no references in any of the stories produced.

## 4. Discussion

The results in the present study aligned with previous research indicating that collaborative storytelling can encourage prosocial behaviors and shared representations when the group works collaboratively [27,38,40,41,42,73]. Therefore, it can be a valuable tool in both face-to-face and distance education [29,74,75,76], especially in coping with social distancing during a global pandemic such as the 2020 SARS-CoV-2 home confinement.

Collaboration, storytelling, and learning require certain conditions to occur effectively. Therefore, integrating technology in the primary education context needs certain conditions to be met. Specifically, collaborative interaction requires technology to adapt to the context’s needs. Otherwise, it could become a barrier to collaboration and learning [30,77]. Results confirmed the first hypothesis, revealing that students in primary school can use an online platform and collaboratively create stories in a 100% online structured environment with facilitator arbitration. The limitations in digital autonomy found in the first years of primary school can be managed by modifying the level of digital autonomy required to carry out the sessions.

Regarding the formal aspects of the stories, the quality of the stories concerning the school grade is distinguished only in terms of their length, whereas the students in the lower grades created longer stories than in higher grades. It means that in higher grades, students needed fewer words to create a story with a comparable structure and cohesion to the longer stories created by younger students. This result shows significant differences between the methodology of storytelling. In sessions involving the chained-stories methodology, there were more participants in each session, and the nature of the turn-based participation in creating the story may have influenced the length of the final story.

On the other hand, there are no differences between grades or methodologies in story structure and narrative cohesion at reasonable levels for all grades. The structure and cohesion of the stories can be a potential benefit of collaboration between students. In other words, group work may have favored creating more sophisticated stories than creating stories individually, according to what is expected for the different age groups [2,7].

As for the stories’ content, the groups’ stories are balanced in all the indexes. Balance in story content appears primarily related to characters’ behavior and relationships, while story problems were independent of the session topics. Collaborative work may have favored the creation of balanced stories. The need for group agreement of the final story in this study may have influenced the decision-making process, where peer contribution acted as a mutual form of control and regulation of the declared story plots, helping group members to close previous elements opened throughout the story [24,25,26,78].

Finally, the analysis of COVID-19 references in the stories reflects a total frequency of appearance of 15% of the 81 stories prepared by the children. We observed a decrease in references from April to June 2020, with April being the month where most references were found. This decrease in references can be related to students’ progressive normalization of the pandemic situation and the fact that, as of May 2020, the community of Madrid began to allow children to leave their homes, and children may have reduced their concerns about the lockdown situation in this context. Additionally, the lower frequency of COVID-19 references could be indicative of both a lower emotional strain and, therefore, a lower need to express concerns or discomfort related to the pandemic, as well as a lower capacity or habit to share negative emotions and concerns within the group, during an enjoyable shared activity.

### Limitations

As for the study limitations, we launched the online collaborative storytelling activity due to the 2020 SARS-COV2 pandemic schools’ closure in Spain. It initially involved intense coordination and organization between teachers and families practically simultaneously at the start of the activity. This may have influenced attendance, quality, and organization during the first two weeks of activity.

The different group sizes and topics per grade are due to the voluntary nature of the extra-curricular activity, which did not require regular attendance. It may have influenced the final benefit of the activity for participants and the comparability of data between grades. A more even distribution of participants from each grade in attendance would increase the proportionality in the data.

In addition, the irregular attendance of students added to the difficulty of creating stable work groups. In future studies, it will be necessary to ensure more stability of the working groups to carry out a more-specific and detailed observation of the behavioral and relational evolution within the collaborative group and the progress of the stories created.

## 5. Conclusions

The study indicates that primary students can work collaboratively in an online digital storytelling activity during a pandemic crisis, with some methodology adaptations to 1st and 2nd grade, because of a more limited digital autonomy. Online digital storytelling enhances the possibility of creating shared stories and their subsequent transmission through collaborative communities where students are the protagonists in real-time interactions. It can be supported by a flexible and coordinated pedagogical organization, an open-source learning-management system such as Moodle, and several video-call rooms based on the Google Meet technology. The enduring values of narrative structure and cohesion in different grades, the positive content balance of the stories, and the few references to the SARS-CoV-2 pandemic indicate that online cooperative storytelling can provide cognitive, emotional, and social support in primary school. The form and content of a story may change from person to person and from group to group. Still, it reflects the narrator’s linguistic, symbolic, emotional, experiential, and decision-making abilities. When a collaborative group creates a story, group members need to agree on what and how to tell it. This agreement implies a process of decision-making, listening, and consensus that allows the regulation and the shared construction of the general plan of the story. When a consensus is reached, participants’ contributions help to control and regulate the arguments stated throughout the story, closing elements previously opened by a groupmate. In this way, collaboration allows students to reach a higher story level than different group members could achieve alone. Finally, the objective of preventive programs and tailored interventions should be to implement educational policies aimed at supporting children and families during a pandemic crisis. Moreover, future studies will also be essential to explore students’ skills development through this kind of storytelling activity.

## Figures and Tables

**Table 1:** Participants (N = 116).

Grade	Age in Months		Gender		Students		Difficulty Type		Total
	Range	Mean (SD)	Female	Male	No Diff.	With Diff.	Neurodev. ${}^{1}$	Funct. ${}^{2}$	
1st	76–85	80 (3)	14 (12.00%)	13 (11.20%)	26 (22.40%)	1 (0.80%)	0 (0.00%)	1 (0.80%)	27 (23.20%)
2nd	88–99	93 (3)	23 (19.80%)	16 (13.80%)	36 (31.00%)	3 (2.60%)	1 (0.80%)	2 (1.70%)	39 (33.60%)
3rd	101–111	106 (2)	8 (6.90%)	4 (3.40%)	11 (9.40%)	1 (0.80%)	1 (0.80%)	0 (0.00%)	12 (10.30%)
4th	115–124	119 (3)	3 (2.60%)	4 (3.40%)	4 (3.40%)	3 (2.60%)	2 (1.70%)	1 (0.80%)	7 (6.00%)
5th	125–135	129 (3)	15 (12.90%)	16 (13.80%)	29 (25.00%)	2 (1.70%)	1 (0.80%)	1 (0.80%)	31 (26.70%)
Total	76–135	103 (19)	63 (54.30%)	53 (45.70%)	106 (91.40%)	10 (8.60%)	5 (4.30%)	5 (4.30%)	116 (100.00%)

${}^{1}$ Neurodevelopmental disorders, including ADHD, communication disorders, and specific learning disorders. ${}^{2}$ Functional difficulties, including emotional and behavioural difficulties.

**Table 2:** Number of group members per grade (116 students; 81 group stories).

Grade	Number of Group Members											Number of Group/Stories
	**2**	**3**	**4**	**5**	**6**	**7**	**8**	**9**	**10**	**15**	**20**	
1st	1	-	-	1	3	3	2	1	-	-	1	12
2nd	-	-	-	1	6	5	3	3	1	1	-	20
3rd	-	1	-	1	1	2	-	1	-	-	-	6
4th	1	5	2	2	1	1	1	1	-	-	-	14
5th	-	2	3	7	6	5	3	2	1	-	-	29
Total	2	8	5	12	17	16	9	8	2	1	1	81

**Table 3:** Kruskal–Wallis test for platform use and collaboration between grades (1st to 5th grade).

	Kruskal–Wallis	df	Asymp. Sig.
Mute a partner	9.33	4	*p* = 0.053
Interrupt	21.76	4	*p* < 0.01
Off topic chat use	10.15	4	*p* = 0.05
Group self-regulation	13.87	4	*p* < 0.01
Positive comments	15.50	4	*p* < 0.01
Task-focused group	13.68	4	*p* < 0.01
Social conscience	35.72	4	*p* < 0.01
Social cognition	32.62	4	*p* < 0.01
Social communication	22.33	4	*p* < 0.01
Social motivation	19.27	4	*p* < 0.01
Space for everyone	27.61	4	*p* < 0.01
Inclusion	26.70	4	*p* < 0.01

**Table 4:** Spearman’s r correlation between course grade (1st to 5th), number of group members, platform use, and collaboration.

	GM	SIL	INT	CUT	GSR	PC	TFG	SCS	SC	SCM	SM	SE	INC
Course Grade (CG)	−0.26 *	−0.14	−0.02	−0.03	−0.07	0.02	−0.04	−0.03	−0.02	−0.01	0.13	−0.10	0.05
N. group members (GM)		0.08	0.04	0.20	0.13	−0.11	−0.12	−0.16	−0.12	−0.04	−0.18	−0.15	−0.20
Silence (SIL)			0.46 **	0.21	0.21	−0.25 *	−0.54 **	−0.30 *	−0.36 **	−0.47 **	−0.34 **	−0.40 **	−0.35 **
Interrupt (INT)				0.34 **	0.04	−0.35 **	−0.57 **	−0.59 **	−0.48 **	−0.57 **	−0.29 *	−0.46 **	−0.47 **
Chat use off topic (CUT)					−0.02	−0.18	−0.18	−0.43 **	−0.35 **	−0.28 *	−0.25 *	−0.32 **	−0.31 **
Group self-regulation (GSR)						0.27 *	−0.15	−0.01	0.03	0.02	0.12	0.02	0.01
Positive comments (PC)							0.32 **	0.51 **	0.40 **	0.46 **	0.36 **	0.49 **	0.50 **
Task-focused group (TFG)								0.53 **	0.42 **	0.51 **	0.46 **	0.56 **	0.51 **
Social conscience (SCS)									0.81 **	0.74 **	0.59 **	0.63 **	0.53 **
Social cognition (SC)										0.70 **	0.51 **	0.52 **	0.52 **
Social communication (SCM)											0.62 **	0.67 **	0.62 **
Social motivation (SM)												0.55 **	0.48 **
Space for everyone (SE)													0.82 **
Inclusion (INC)													

* *p* < 0.05; ** *p* < 0.01

**Table 5:** Descriptive statistics for balance indexes (N = 81).

	Min; Max	Mean (SD)	Skeweness	Kurtosis
Balance 1	−3.00; 6.00	1.32 (1.90)	−0.32 (0.27)	−0.06 (0.53)
Balance 2	−5.00; 6.00	1.89 (1.87)	−0.46 (0.27)	1.82 (0.53)
Balance 3	−11.00; 8.00	0.73 (2.67)	−0.57 (0.27)	4.06 (0.53)
Balance 4	−11.00; 4.00	−0.59 (2.47)	−1.30 (0.27)	3.07 (0.53)

**Table 6:** Non-parametric test (Kruskal–Wallis and Mann–Whitney test) for history content-balance indexes between grades (1st to 5th grade), number of group members, and methodologies (story chain vs. small group story).

	Grades (1st to 5th Grade) Kruskal–Wallis Test	Group Members Kruskal–Wallis Test	Methodologies (Story Chain vs. Small Group Story) Mann–Whitney Test
Balance 1	6.39; df 4; *p* = 0.17	15.32; df 10; *p* = 0.12	U = 836.50; *p* = 0.66
Balance 2	6.97; df 4; *p* = 0.14	11.77; df 10; *p* = 0.30	U = 803.50; *p* = 0.91
Balance 3	9.41; df 4; *p* = 0.05	12.17; df 10; *p* = 0.27	U = 821.50; *p* = 0.77
Balance 4	4.45; df 4; *p* = 0.35	16.06; df 10; *p* = 0.09	U = 648.50; *p* = 0.16

**Table 7:** Frequency of references to COVID-19 or the pandemic per grade (N = 81).

Grade	1st	2nd	3rd	4th	5th	Total
Number of stories	12	20	6	14	29	81
Direct reference to COVID-19/Pandemic	2	0	0	2	1	5 (6.17%)
Indirect reference to COVID-19/pandemic—catastrophe	1	0	1	1	0	3 (3.70%)
Indirect reference to COVID-19/pandemic—illness	0	2	1	0	1	4 (4.93%)
Total references to COVID-19/pandemic	3	2	2	3	2	12 (14.81%)

## Data Availability

Data are fully available here: https://figshare.com/s/ea1525fdda97834d4cb4 (accessed on 20 November 2021).

## Supplementary Materials

The following are available online at https://www.mdpi.com/article/10.3390/ejihpe11040115/s1, Table S1: Story 34, 1st grade with direct reference to COVID-19 theme; Table S2: Story 10, 5th grade with indirect (illness) reference to COVID-19 theme; and Table S3: Descriptive Statistics (116 students; 71 sessions; 81 stories).
