# Supplementary material for: Children’s Online Collaborative Storytelling during 2020 COVID-19 Home Confinement
Source: Eur J Investig Health Psychol Educ. 2021 Dec 10;11(4):1619–34. doi: 10.3390/ejihpe11040115 (PMC8700547; doi:10.3390/ejihpe11040115)
Supplement: Supplementary file 1 [file ejihpe-11-00115-s001.zip › ejihpe-1464747-supplementary.pdf]

Table S1: Story 34, 1st grade with direct reference to COVID-19 theme

Story ID: 34;
Grade: 1st grade.
Session: 5.
Number of participants: 9;
Total participants with difficulties: 0;
Storytelling methodology: chained story.
Session theme: Family.
Date: 28/04/2020.
COVID-19 theme references: 2 (direct). Propositions: //…//. Episodes: […].
Cohesion index: 5 (Thematic sequences).
Structure index: 4.

COHESION INDEX: 5 (Thematic sequences). Children insert an abstract element from this level (5) that interconnects at least two story episodes. This abstract element can include a theme, a conflict between characters, or a problem. Level 5 is essentially a level 4 in which the child inserts a theme, a point of view (rather abstract) that organizes the story. At this level, children get a sign of conflict, danger, and threat in the story without a clear protagonist and antagonist. The opposing forces of history are not well developed (for example, "a monster comes"). Maybe it is necessary to read "between the lines of the story" to identify a conflict, a threat, a problem.

STORY (the number of story words can vary due to Spanish-English translation): [Once upon a time, there was a little she-bear] [and the little he-bear went to play in the yard.] [Later, the she-bear went to the doctor// because she had fallen off the slide// and broke her leg//], [and the she-bear girl got better.] [Then they come back home// and played], [then they went outside again// and jumped down the slide]. [Then the father was going to scare the little he-bear // and he scared him,// and when the little he-bear got scared// he fell// and hurt his knee,] [and the little he-bear went to the doctor// and found the policeman,// he found the policeman in the doctor// because the policeman had broken his arm.] [Then the doctor dropped a medicine on his foot// and hurt his foot]. [Then, since the mother was prankish// she scared the father //and then the father broke his arm too]. [Then the rabbit came,// then it happened to him that he had broken his leg] [and thank goodness that the mother had studied medicine// and she began to cure her daughter and her son]. [Then the duck came// and ran into the mother's leg// and the duck also broke an arm.] [Later, the bunny didn't know what happened// and went to the doctor// to see what happened to the others.] [So, the cow was going to her house// and get sick of coronavirus.] [The father was cured] [and then luckily, he was also studying a little veterinarian// and he cured the duck and the cow]. [And the rabbit, when he had already been cured by the father// broke his tail.] [Dad fell out of the chair// and broke his back.] [Then the doctor came// and cured everyone], [then the doctor who had cured everyone hurt himself// because he fell]. [And then while dad healed// while he was on the computer// he broke down and hit his eye.] [Then, as the children were cured,// the mother gave them some gifts// because they had been delicious.] [Then the duck had come// and the duck had become a doctor// and she cured them.] [After, when they all were healed,// they all played together.] [They bought bread// and gave him the little frog// that was in the pond,] [they went on holidays,// so when they come back from holidays // they played with the sand.] [When they went swimming,// a crab bit the brother's toe] [and the nurse dropped a medicine cabinet containing all the viruses// and they became infected.] [Then all the zoo animals escaped from the zoo,// then when they saw that the animals escaped// they put them all inside] [and then they went back to the beach// and dived,// and they collided] [and went to the doctor again.] [There was a magical bird// that gave them magical powers// that they would always be happy] [and then with the bird's powers// they were cured] [and come back to the beach.]

Platform Use	Mute a Partner	1
	Interruptions	1
	Off-topic chat use	1
	Group self regulation	3
	Positive comments	3
Collaboration	Focus on the task	3
	Social awareness	3
	Social cognition	3
	Social motivation	3
	Space for everyone	3
	Inclusion	3

Story words, Propositions & Episodes	Number of words	509
	Propositions	85
	Episodes	35
Story Structure and Cohesion	Narrative structure	4
	Narrative cohesion	5
Story content balance indexes	Balance 1	6
	Balance 2	1
	Balance 3	2
	Balance 4	-1
Covid-19 related content (0-1)	Direct ref.	1
	Indirect ref (catastrophe)	0
	Indirect ref. (illness)	0

Table S2: Story 10, 5th grade with indirect (illness) reference to COVID-19 theme

Story ID: 10;
Grade: 5th grade.
Number of participants: 5;
Total participants with difficulties: 0;
Storytelling methodology: little group.
Session theme: Family.
Date: 28/04/2020.
COVID theme references: 1 (indirect - illness). Propositions: //…//. Episodes: […].
Cohesion index 8 (Dyadic structure with intermediate problem-solution events).
Structure index: 4

COHESION INDEX: 8 (Dyadic structure with intermediate problem-solution events). In the story, children introduce a problem-solution dyad with an event between the problem and its solution. An intermediate problem-solution element is an event where the actions of one or more characters lead to solving or complicating the initial problem. Actions that can be part of an intermediate problem-solution event are, for example, "asking for and giving help," "calling the policeman," etc.

STORY (the number of story words can vary due to Spanish-English translation): [Once there were four bears// who couldn't cook], [and once they had a party in their area// and they had to bring food//] [and then they had to buy food,//but since it was illegal in this area//, the police arrested them// and told them// that they had to cook themselves,// taking them to the police station.] [they let them go// but told them they had to prepare their food//so they prepared the food] [and when people ate it// they all started vomiting// and had to call the doctor]. [The doctor cured them with rabbit soup// and they were healed,] [but after this traumatic experience// they decided never to cook again].

Platform Use	Mute a Partner	1
	Interruptions	2
	Off-topic chat use	1
	Group self regulation	2
	Positive comments	1
Collaboration	Focus on the task	2
	Social awareness	2
	Social cognition	3
	Social motivation	3
	Space for everyone	3
	Inclusion	3
Story words, Propositions & Episodes	Number of words	113
	Propositions	21
	Episodes	7
Story Structure and Cohesion	Narrative structure	4
	Narrative cohesion	8
Story content balance indexes	Balance 1	2
	Balance 2	4
	Balance 3	0
	Balance 4	0
Covid-19 related content (0-1)	Direct ref.	0
	Indirect ref (catastrophe)	0
	Indirect ref. (illness)	1

Table S3: Descriptive Statistics (116 students; 71 sessions; 81 stories

	Grade		1st	2nd	3rd	4th	5th
	Number of stories		12	20	6	14	29
STORY FORM	Number of words	M (SD)	402.41 (119.52)	289.65 (138.37)	444.00 (138.93)	337.57 (135.50)	221.31 (86.00)
		Min; Max	217; 587	97; 676	210; 586	120; 648	78; 427
		Shapiro Wilk	p = 0.88	p = 0.16	p = 0.35	p = 0.85	p = 0.63
	Propositions	M (SD)	61.25 (18.32)	46.65 (20.47)	73.00 (25.559	51.14 (16.90)	33.14 (13.79)
		Min; Max	33; 85	18; 101	25; 92	23; 85	10; 63
		Shapiro Wilk	p = 0.45	p = 0.28	p = 0.03	p = 0.76	p = 0.28
	Episodes	M (SD)	21.0 (6.71)	15 (5.73)	22.17 (7.11)	13.93 (4.65)	11.41 (4.82)
		Min; Max	14; 35	7; 29	9; 30	8; 24	4; 27
		Shapiro Wilk	p = 0.50	p = 0.49	p = 0.38	p = 0.31	p <.01
	Narrative structure	M (SD)	4.25 (.75)	4.15 (.93)	4.17 (.98)	4.64 (.93)	4.00 (1.07)
		Min; Max	3; 6	3; 6	3; 5	3; 6	2; 6
		Shapiro Wilk	p = 0.04	p = 0.01	p = 0.12	p = 0.05	p < 0.01
	Narrative cohesion	M (SD)	8.25 (2.09)	8.25 (1.68)	7.83 (2.23)	9.14 (0.95)	8.10 (1.52)
		Min; Max	5; 11	5; 11	6; 11	8; 11	6; 11
		Shapiro Wilk	p = 0.25	p = 0.23	p = 0.04	p < 0.01	p < 0.01
STORY CONTENT	Problems positively solved	M (SD)	3.17 (2.12)	1.80 (1.79)	1.00 (1.09)	3.43 (2.34)	2.45 (1.72)
		Min; Max	0; 8	0; 8	0; 3	0; 7	0; 6
		Shapiro Wilk	p = 0.63	p <.01	p < 0.01	p = 0.25	p = 0.22
	Problems negatively solved	M (SD)	1.75 (1.71)	2.40 (2.46)	1.17 (1.17)	1.31 (1.60)	1.46 (1.83)
		Min; Max	0; 4	0; 11	0; 3	0; 5	0; 7
		Shapiro Wilk	p = 0.03	p < 0.01	p = 0.42	p < 0.01	p < 0.01
	Problems without solution	M (SD)	2.67 (1.97)	2.05 (1.54)	1.67 (0.82)	2.43 (1.28)	1.62 (1.01)
		Min; Max	0; 6	0; 5	1; 3	0; 4	0; 3
		Shapiro Wilk	p = 0.39	p = 0.02	p = 0.31	p = 0.13	p <.01
	Total problems	M (SD)	1.08 (1.24)	1.10 (1.16)	1.00 (1.26)	0.57 (.85)	0.93 (1.25)
		Min; Max	0;4	0; 3	0; 3	0; 3	0; 4
		Shapiro Wilk	p = 0.03	p < 0.01	p = 0.04	p <.01	p < 0.01
	Positive relationships between characters	M (SD)	1.67 (1.37)	1.05 (.76)	1.00 (.00)	0.29 (.47)	0.83 (.97)
		Min; Max	0; 5	0; 3	1; 1	0; 1	0; 3
		Shapiro Wilk	p = 0.02	p < 0.01	p < 0.01	p < 0.01	p < 0.01
	Negative relationships between characters	M (SD)	5.00 (2.80)	3.95 (1.85)	3.50 (1.38)	3.14 (1.10)	2.79 (1.45)
		Min; Max	0; 10	1; 8	2; 5	1; 5	0; 7
		Shapiro Wilk	p < 0.01	p = 0.32	p = 0.42	p = 0.24	p = 0.06
	Characters' adaptive behaviors	M (SD)	2.08 (1.88)	1.90 (1.55)	1.67 (1.03)	2.00 (1.17)	1.52 (.95)
		Min; Max	0; 6	0; 5	0; 3	0; 4	0; 3
		Shapiro Wilk	p = 0.04	p = 0.01	p = 0.32	p = 0.14	p < 0.01
	Characters' aggressive behavior	M (SD)	1.83 (1.95)	2.20 (2.55)	2.50 (2.34)	1.57 (1.45)	1.24 (1.62)
		Min; Max	0; 6	0; 12	0; 6	0; 4	0; 7
		Shapiro Wilk	p = 0.16	p < 0.01	p = 0.42	p = 0.03	p < 0.01
	Characters' rule-rejecting behaviors	M (SD)	0.42 (.99)	0.50 (1.10)	1.17 (1.17)	0.50 (1.09)	0.41 (.63)
		Min; Max	0; 3	0; 4	0; 3	0; 4	0; 2
		Shapiro Wilk	p < 0.01	p < 0.01	p = 0.31	p < 0.01	p < 0.01
	Characters' guilt-ridden behaviors	M (SD)	0.18 (.40)	0.50 (.22)	0.50 (.84)	0.07 (.27)	0.21 (.62)
		Min; Max	0; 1	0; 1	0; 2	0; 1	0; 3
		Shapiro Wilk	p < 0.01	p < 0.01	p < 0.01	p < 0.01	p <.01
	Balance 1. positive vs negative	M (SD)	1.75 (2.45)	1.05 (2.11)	0.67 (1.75)	2.28 (1.26)	1.00 (1.67)
		Min; Max	-3; 6	-3; 5	-2; 3	0; 4	-3; 3

	problems solution	Shapiro Wilk	p = 0.91	p = 0.44	p = 0.92	p = 0.25	p < 0.01
	Balance 2. solved vs not-solved problems	M (SD)	2.17 (2.25)	1.75 (2.47)	0.50 (1.87)	2.71 (1.20)	1.76 (1.33)
		Min; Max	-1; 6	-5; 6	-3; 2	1; 4	-1; 4
		Shapiro Wilk	p = 0.54	p = 0.20	p = 0.22	p = 0.03	p = 0.02
	Balance 3. positive vs negative relationships	M (SD)	1.42 (2.47)	-0.70 (3.03)	-0.17 (1.72)	1.71 (2.58)	1.14 (2.37)
		Min; Max	-1; 8	-11; 4	-3; 2	-3; 7	-4; 6
		Shapiro Wilk	p < 0.01	p <.01	p = 0.49	p = 0.99	p = 0.23
	Balance 4. adaptive vs non adaptive behavior	M (SD)	-0.33 (2.60)	-0.85 (3.06)	-2.50 (2.88)	-0.07 (2.09)	-0.38 (1.97)
		Min; Max	-6; 2	-11; 4	-7; 1	-3; 3	-5; 2
		Shapiro Wilk	p = 0.09	p < 0.01	p = 0.75	p = 0.08	p = 0.14
PLATFORM USE	Mute a partner	M (SD)	1.33 (0.65)	1.50 (0.69)	1.0 (0.00)	1.0 (0.00)	1.31 (0.71)
		Min; Max	1; 3	1; 3	1; 1	1; 1	1; 3
		Shapiro Wilk	p < 0.01	p < 0.01	p < 0.01	p <.01	p <.01
	Interrupt	M (SD)	1.08 (0.29)	1.75 (0.64)	1.0 (0.00)	1.0 (0.00)	1.45 (0.74)
		Min; Max	1; 2	1; 3	1; 1	1; 1	1; 3
		Shapiro Wilk	p < 0.01	p < 0.01	p < 0.01	p <.01	p <.01
	Off topic chat use	M (SD)	1 (0.00)	1.55 (0.76)	1.17 (0.41)	1.07 (0.27)	1.24 (0.57)
		Min; Max	1; 1	1; 3	1; 2	1; 2	1; 3
		Shapiro Wilk	p < 0.01	p < 0.01	p < 0.01	p <.01	p <.01
	Group self-regulation	M (SD)	2 (0.85)	1.40 (0.60)	1.17 (0.41)	1.07 (0.27)	1.58 (0.68)
		Min; Max	1; 3	1; 3	1; 2	1; 2	1; 3
		Shapiro Wilk	p = 0.02	p < 0.01	p < 0.01	p < 0.01	p <.01
	Positive comments	M (SD)	2.42 (0.67)	1.60 (0.68)	2.17 (0.98)	2.5 (0.52)	2.03 (0.68)
		Min; Max	1; 3	1; 3	1; 3	2; 3	1; 3
		Shapiro Wilk	p < 0.01	p < 0.01	p = 0.04	p < 0.01	p < 0.01
COLLABORATION	Focus on the task	M (SD)	2.75 (0.45)	2.35 (0.49)	3.00 (0.00)	2.86 (0.36)	2.41 (0.73)
		Min; Max	2; 3	2; 3	3; 3	2; 3	1; 3
		Shapiro Wilk	p < 0.01	p < 0.01	p < 0.01	p <.01	p <.01
	Social awareness	M (SD)	3.00 (0.00)	1.85 (0.67)	2.66 (0.52)	3.00 (0.00)	2.41 (0.63)
		Min; Max	3; 3	1; 3	2; 3	3; 3	1; 3
		Shapiro Wilk	p < 0.01	p < 0.01	p < 0.01	p <.01	p <.01
	Social cognition	M (SD)	3.00 (0.00)	1.90 (0.72)	2.33 (0.52)	3.00 (0.00)	2.45 (0.63)
		Min; Max	3; 3	1; 3	2;3	3; 3	1; 3
		Shapiro Wilk	p < 0.01	p < 0.01	p < 0.01	p <.01	p <.01
	Social communication	M (SD)	3.00 (0.00)	2.10 (0.85)	3.00 (0.00)	2.93 (0.27)	2.65 (0.48)
		Min; Max	3; 3	1; 3	3; 3	2; 3	2; 3
		Shapiro Wilk	p < 0.01	p < 0.01	p < 0.01	p <.01	p <.01
	Social motivation	M (SD)	2.75 (0.45)	2.20 (0.52)	2.83 (0.41)	2.93 (0.27)	2.62 (0.56)
		Min; Max	2; 3	1; 3	2; 3	2; 3	1; 3
		Shapiro Wilk	p < 0.01	p < 0.01	p < 0.01	p <.01	p <.01
	Space for everyone	M (SD)	2.92 (0.29)	2.20 (0.61)	3.00 (0.00)	3.00 (0.00)	2.45 (0.57)
		Min; Max	2; 3	1; 3	3; 3	3; 3	1; 3
		Shapiro Wilk	p < 0.01	p < 0.01	p < 0.01	p <.01	p <.01
	Inclusion	M (SD)	2.92 (0.29)	2.20 (0.61)	3.00 (0.00)	3.00 (0.00)	2.65 (0.48)
		Min; Max	2; 3	1; 3	3; 3	3; 3	2; 3
		Shapiro Wilk	p < 0.01	p < 0.01	p < 0.01	p <.01	p <.01
